# Toxicity of nine insecticides on four natural enemies of *Spodoptera exigua*

**DOI:** 10.1038/srep39060

**Published:** 2016-12-13

**Authors:** Yongqiang Liu, Xiangying Li, Chao Zhou, Feng Liu, Wei Mu

**Affiliations:** 1 State Key Laboratory for Biology of Plant Diseases and Insect Pests, Institute of Plant Protection, Chinese Academy of Agricultural Sciences, Beijing 100193, China; 2 Key Laboratory of pesticide Toxicology & Application technique, College of Plant Protection, Shandong Agricultural University, Tai’an 271018, China; 3 Foshan Center for Environmental Health and Safety Assessment, Foshan 528000, China; 4 Taian Academy of Agricultural Sciences, Tai’an 271000, China

## Abstract

*Spodoptera exigua*, which feeds on various crops worldwide, has natural enemies that are susceptible to the insecticides used against *S. exigua*. We investigate the toxicity and residue risk of 9 insecticides on the development of *H. axyridis, C. sinica, S. manilae* and *T. remus. S. manilae* and *T. remus* adults were sensitive to all 9 insecticides (LC_50_ less than 2.75 mg a.i. liter^−1^), while *H. axyridis* and *C. sinica* adults were less sensitive (LC_50_ between 6 × 10^−5^ mg a.i. liter^−1^ and 78.95 mg a.i. liter^−1^). Emamectin benzoate, spinosad, indoxacarb, alpha-cypermethrin, chlorfenapyr and chlorantraniliprole showed no toxicity on *H. axyridis, C. sinica, S. manilae* and *T. remus* pupae with the recommended field concentrations. The risk analysis indicated that chlorantraniliprole is harmless to larvae of four natural enemies and adult of *H. axyridis, C. sinica* and *S. manilae*. Emamectin benzoate and spinosad had higher safety to the development of *H. axyridis, C. sinica, S. manilae* and *T. remus* with the risk duration less than 4d. Indoxacarb, tebufenozide, chlorfenapyr, methomyl, alpha-cypermethrin and chlorpyrifos showed dangerously toxic and long risk duration on *S. manilae* and *T. remus* adults.

The beet armyworm, *Spodoptera exigua* (Hübner) (Lepidoptera: Noctuidae), is a polyphagous insect feeding on various agricultural crops including vegetables, cotton, and ornamentals^1^. They have spread rapidly in the tropical and sub-tropical regions, due to their migratory habit and world-wide distribution of available host plants^2^,^3^. The use of insecticides remains the major strategy in dealing with *S. exigua* as it is quick, cost-effective and effective. However, lethal and sub-lethal effects of broad-spectrum insecticides may impact beneficial species^4^,^5^. Misuse of insecticides might account for pest outbreaks because extensive and intensive insecticides applied to target pests and may accelerate resistance development. Another important factor is that insecticides may indiscriminately kill natural enemies. In fact, natural enemies tend to be more susceptible to insecticides than pests^6^,^7^.

Insecticides and biological control are two important management strategies. Integrating these two strategies is important for the success of any management program^8^. Insecticides should be only used when necessary and be least disruptive to biological control^9^. Knowing the impact of insecticides on natural enemies is essential for integration of these two strategies^10^. It is essential to understand the dynamics of integrating prudently used chemical pesticides along with biological control organisms for the effective implementation of an IPM strategy^8^,^10^.

*Harmonia axyridis* (Pallas), (Coleoptera: Coccinellidae) and *Chrysoperla sinica* (Tjeder), (Neuroptera: Chrysopidae) are predator with a wide host range, attacking aphids, eggs and larvae of Lepidoptera including *S. exigua*, the two predators are an important component of natural and agricultural habitats^11^,^12^*. Snelleniua manilae* (Ashmead) and *Telenomus remus* Nixon (Hymenoptera: Scelionidae) are larvae and egg parasitoid, respectively, both *S. manilae* adults and *T. remus* adults have control ability against *S. exigua*^13^,^14^,^15^,^16^. Two predators and two parasitoids have potential as biological agents for *S. exigua* suppression in the field.

However, insecticide use can greatly suppress the populations of natural enemies, lowering their biological control potential^17^,^18^. Presently, relatively little is known about the susceptibility of four natural enemies to currently popular insecticides, which are being investigated as potential alternatives to organophosphate insecticides. Information on the relative toxicity of various insecticides to four natural enemies *H. axyridis, C. sinica, S. manilae* and *T. remus* can aid in the development of an integrated pest management strategy for *S. exigua.*

## Results

### Toxicity to three *H. axyridis* developmental life stages

The LC_50_ values were significantly different for different developmental life stages. For *H. axyridis* larvae, The LC_50_ values of selected insecticides to *T. nubilale* ranged from 0.015 to 13.31 mg a.i. liter^−1^. The order of toxicity (high-low) for the 9 insecticides was as follows: methomyl > indoxacarb > tebufenozide > alpha-cypermethrin > chlorpyrifos > chlorantraniliprole, spinosad ≥ chlorfenapyr, emamectin benzoate (LC_50_ values with overlapping the 95% confidence intervals were classified as having the same level of toxicity) (Table 1). For *H. axyridis* pupae there was no significant lethal effect at the recommended field application rate of the insecticides except chlorpyrifos, tebufenozide and methomyl (LC_50_: 527.68, 350.08 and 314.75 mg a.i. liter^−1^, respectively) (Table 3). For adult *H. axyridis*, chlorpyrifos displayed the highest toxicity (LC_50_: 0.04 mg a.i. liter^−1^ at 24 h). Tebufenozide, chlorfenapyr and alpha-cypermethrin displayed higher toxicity (LC_50_ between 0.48 and 1.91 mg a.i. liter^−1^, respectively). Spinosad, indoxacarb, methomyl, chlorantraniliprole and emamectin benzoate had slower toxicity (LC_50_ between 16.3 and 22.52 mg a.i. liter^−1^) (Table 1).

### Toxicity to three *C. sinica* developmental life stages

The insecticides didn’t show lethal effect to *C. sinica* larvae 24 h after treatment at the recommended field application concentrations except chlorpyrifos (LC_50_: 185.67 mg a.i. liter^−1^) and methomyl (LC_50_: 162.08 mg a.i. liter^−1^) (Table 1), and the percentage emergence rate of *C. sinica* pupae at the recommended field rate of the insecticides was not significantly different from untreated *C. sinica* pupae (*F* = 0.65, d.f. = 9, *P* = 0.74) (Table 2). For *C. sinica* adult, the order of toxicity (high-low) of the 9 insecticides was as follows: chlorfenapyr > alpha-cypermethrin, methomyl > indoxacarb > emamectin benzoate > chlorpyrifos > spinosad, chlorantraniliprole ≥ tebufenozide. Chlorfenapyr displayed the highest toxicity (LC_50_: 6 × 10^−5^ mg a.i. liter^−1^), while tebufenozide had the lowest toxicity (LC_50_: 29.09 mg a.i. liter^−1^) (Table 1).

### Toxicity to three *S. manilae* developmental life stages

Concentrations treated parasitized *S. exigua* larvae were based on the LC_50_ value for *S. exigua*. The percentage pupation rate of *S. manilae* larvae by treated parasitized *S. exigua* larvae was not significantly different from untreated *S. manilae* larvae (*F* = 0.28, d.f. = 9, *P* = 0.97) (Table 3). The percentage emergence rate of *S. manilae* pupae at the recommended field rate of the insecticides was not significantly different from untreated *S. manilae* pupae (*F* = 0.30, d.f. = 9, *P* = 0.97) (Table 2). All insecticides were toxic to *S. manilae* adults 24 h post-treatment (LC_50_ values between 1 × 10^−5^ and 2.75 mg a.i. liter^−1^). The order of toxicity (high-low) of the 9 insecticides was as follows: methomyl > alpha-cypermethrin > tebufenozide > emamectin benzoate > spinosad > chlorfenapyr > indoxacarb, chlorpyrifos > chlorantraniliprole (Table 1).

### Toxicity to three *T. remus* developmental life stages

None of the insecticides affected *T. remus* larvae survival (Table 1). The percentage emergence rate of *T. remus* pupae at the recommended field rate of the insecticides was not significantly different from untreated *T. remus* pupae (*F* = 0.16, d.f. = 9, *P* = 0.99) (Table 2). All insecticides were toxic to *T. remus* adults 24 h post-treatment (LC_50_ values less than 1 × 10^−5^ mg a.i. liter^−1^).

### Risk assessment of insecticides on *H. axyridis, C. sinica* and *T. remus* larvae and *H. axyridis, C. sinica, S. manilae* and *T. remus* adult

The classification of the 9 insecticides based on risk quotient values in presented in Table 1. All tested insecticides were safe for larvae of *C. sinica* and *T. remus* with risk quotients less than 1. Emamectin benzoate, spinosad, alpha-cypermethrin, chlorfenapyr and chlorantraniliprole were safe for *H. axyridis* larvae (risk quotients, 0.17–36.89), while emamectin benzoate, spinosad, indoxacarb, alpha-cypermethrin, chlorantraniliprole and methomyl were safe for *H. axyridis* adult (risk quotients, 0.028–5.13). However, methomyl (risk quotient, 17500.00) and chlorpyrifos (risk quotient, 15000.00) was considered dangerous to *H. axyridis* larvae and adult, respectively, whereas chlorpyrifos, indoxacarb and tebufenozide were slightly to moderately toxic to *H. axyridis* larvae (risk quotients, 291.26–1442.31), and chlorfenapyr and tebufenozide were slightly to moderately toxic to *H. axyridis* adult (risk quotients, 76.53–390.63). For *S. manilae* and *T. remus* adult, all tested insecticides were dangerously toxic, except indoxacarb (risk quotient, 267.86) which was slightly to moderately toxic, and chlorantraniliprole (risk quotient, 8.18) was safe to *S. manilae* adult. For *C. sinica* adult, emamectin benzoate, spinosad, tebufenozide and chlorantraniliprole were safe with risk quotients of 0.66–5.49, indoxacarb and chlorpyrifos (risk quotients, 775.86 and 952.38, respectively) were slightly to moderately toxic, whereas alpha-cypermethrin, chlorfenapyr and methomyl (risk quotients, 12430.94–1.33 × 10^5^) were dangerously toxic.

**Figure 1:**
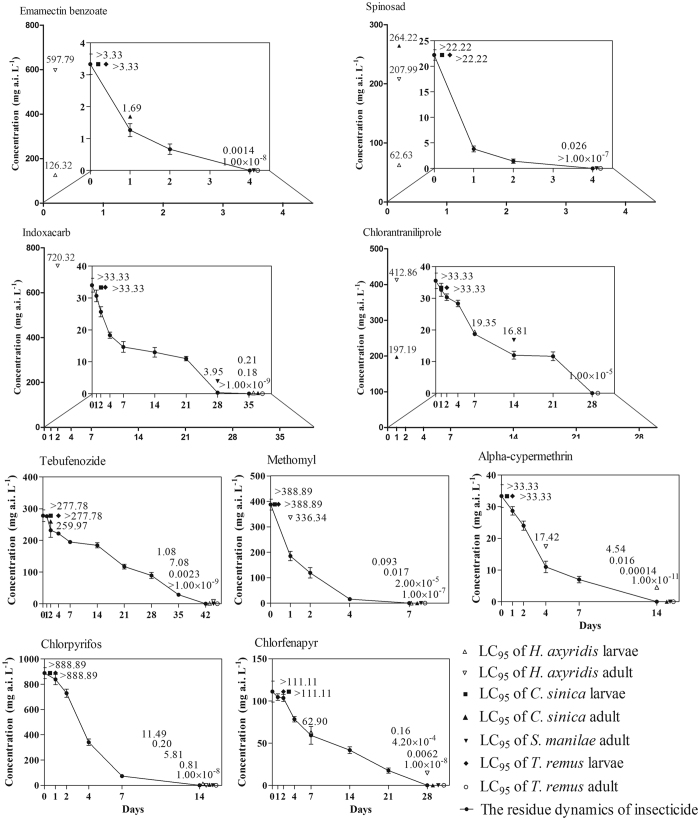
Comparison of LC_95_ value of 9 insecticides to *H. axyridis, C. sinica* and *T. remus* larvae and *H. axyridis, C. sinica, S. manilae* and *T. remus* adult with the residues of the field recommend concentration.

The comparison of LC_95_ values of 9 insecticides to *H. axyridis, C. sinica* and *T. remus* larvae and *H. axyridis, C. sinica, S. manilae* and *T. remus* adult with their field recommended rates is shown in Fig. 1. For *H. axyridis* larvae, the LC_95_ value of emamectin benzoate and spinosad were distinctly higher than its recommended field concentrations, indicating that these insecticide is harmless to the *H. axyridis* larvae. However, the LC_95_ value of tebufenozide was still lower than its residues of the recommended field concentrations occurring 35d after treatment, indicating that tebufenozide had the longest risk duration, followed by indoxacarb (28d), alpha-cypermethrin (7d), chlorpyrifos (7d), methomyl (4d), chlorfenapyr (4d) and chlorantraniliprole (4d). For adult *H. axyridis*, emamectin benzoate, spinosad, indoxacarb and chlorantraniliprole were harmless with the LC_95_ value higher than their recommended field concentrations. Similar to *H. axyridis* larvae, tebufenozide had the longest risk duration (35d) to *H. axyridis* adult, followed by chlorfenapyr (21d), chlorpyrifos (7d), alpha-cypermethrin (2d) and methomyl (1d). For *C. sinica* and *T. remus* larvae, the LC_95_ of 9 insecticides were higher than their recommended field concentrations, indicating that the 9 insecticides are harmless to them. For adult *S. manilae* and *T. remus*, the LC_95_ value of all 9 insecticides were significantly lower than their recommended field concentrations, indicating that these insecticides would be harmful to them. Meanwhile, tebufenozide had the longest risk duration to *S. manilae* and *T. remus* adult (35d), followed by chlorfenapyr (21d), chlorpyrifos (7d), alpha-cypermethrin (7d), methomyl (4d) spinosad (2d) and emamectin benzoate (2d), and the risk duration of chlorantraniliprole and indoxacarb on *S. manilae* adult (7d and 21d, respectively) shorter than *T. remus* adult (21d and 28d, respectively).

## Discussion

Insecticides may kill natural enemies because of their common physiology^9^. Insecticide evaluations on natural enemies should include not only acute toxicity but also residual toxicity^19^. Under laboratory conditions, we tested the toxicity and residue risk of 9 insecticides to the development of *H. axyridis, C. sinica, S. manilae* and *T. remus*. Of the 9 insecticides tested, Indoxacarb, tebufenozide, chlorfenapyr, methomyl, alpha-cypermethrin and chlorpyrifos showed dangerously toxic and long risk duration on *S. manilae* and *T. remus* adults, similar situation was also observed on chlorpyrifos to *H. axyridis* adults and chlorfenapyr, methomyl and alpha-cypermethrin to *C. sinica* adults. therefore, these six insecticides are not suitable for the control of *S. exigua*.

Chlorantraniliprole, the first commercial anthranilic diamide insecticide, is a potent and selective activator of insect ryanodine receptor (RyRs) that are critical for muscle contraction^20^,^21^. Activation of the ryanodine receptors in insects affects uncontrolled release of calcium from internal stores in the sarcoplasmic reticulum, causing unregulated release of internal calcium in the cell and leading to feeding cessation, lethargy, muscle paralysis, and ultimately death of the insect^21^. Among the insecticides evaluated, chlorantraniliprole was dangerously toxic and had a long residual (>21d) activity on *T. remus* adults, however, the insecticide was safe to larvae and adult of *H. axyridis, C. sinica* and *S. manilae*. Brugger *et al*.^22^ reported that chlorantraniliprole had selectivity to the beneficial parasitoid wasps *Aphidius rhopalosiphi, Trichogramma dendrolimi, Trichogramma chilonis, Trichogramma pretiosum, Aphelinus mali, Dolichogenidea tasmanica* and *Diadegma semiclausum*^22^. Its use for *S. exigua* IPM is feasible, and it should be selected according to the target species of the four natural enemies.

Emamectin-benzoate and spinosad were safe to the three life stages of *H. axyridis, C. sinica, S. manilae* and *T. remus* and larvae and pupae of *S. manilae* and *T. remus.* Though emamectin-benzoate and spinosad were dangerously toxic to adults of *S. manilae* and *T. remus*, these two insecticides had short risk duration. This may be caused by emamectin-benzoate and spinosad can penetrate leaf tissues by translaminar movement^23^. It also is important that, Emamectin-benzoate and spinosad are safe to mammals and harmless to other enemies^24^,^25^,^26^,^27^. Therefore, these two insecticides are suitable candidates for suppressing outbreaks of S. exigua.

Our study, *C. sinica, S. manilae* and *T. remus* pupae, survived the recommended field rate, probably because the pupae were shielded from insecticide contact by the cocoon. The results indicate that the insecticides can be applied during the pupae of the natural enemies, as discussed previously^28^,^29^. All the insecticides were non-toxic to *T. remus* larvae in our study, maybe *T. remus* larvae were shielded from insecticide contact. Toxicity of the insecticides to *S. manilae* larvae was likely due to direct host death, because the percentage pupation rate of *S. manilae* larvae by treated parasitized *S. exigua* larvae was not significantly different from untreated *S. manilae* larvae. In the present study, most of the *S. exigua* were killed directly by insecticides so it was not possible to distinguish whether *S. manilae* larvae died directly or because their hosts *S. exigua* larvae were killed.

Release of domesticated natural enemies to control insect has become an important tactic for the management of insect pests in many agricultural crops. Biological control has been increasingly used in crop protection over the past 30 years, with the production of biological control agents also increasing, with more than 130 species of predators and parasitoids on the market in 2000^30^. Laboratory experiment show that *S. manilae* and *T. remus* have good control effect against *S. exigua*^13^,^31^,^32^. The outlook for the success of parasitoids *S. manilae* and *T. remus* against S. *exigua* in crops is now more positive^13^. Due to their strong toxic on *S. manilae* and *T. remus* adults, 8 out of 9 of the insecticides (emamectin-benzoate, spinosad, indoxacarb, tebufenozide, methomyl, alpha-cypermethrin, chlorpyrifos and chlorfenapyr) as measured in this study should be applied with great caution if releasing adults of *S. manilae* and all tested insecticides in this study should be used with great caution when releasing adults of *T. remus*.

The extensive use of insecticides often promotes the development of insect resistance. At present, *S. exigua* have developed high levels of resistance to emamectin benzoate, cypermethrin and chlorpyrifos^33^. Therefore, the high-efficacy insecticides with minimal impact on natural enemies should be used as alternatives. This study showed important results that will help pest managers to choose the best insecticides to be applied, because products with the lowest impact on biological control agents are the most appropriate for use in IPM programs. However, the impact of insecticides on natural enemies is complex, which requires systematic study to determine sublethal effects on the biology, physiology, and behavior of four natural enemies populations.

## Methods

### Insects culture

*H. axyridis* adults and *C. sinica* adults were obtained from a *Brassica oleracea L. var. capitata L* field in Tai’an, China. After collection, they were stored separately in plastic insect boxes (23 cm long × 15 cm wide × 9 cm high) with 20–30 adults per box, and maintained under laboratory conditions of 27 ± 1 °C and a photoperiod of 16:8 h (L:D). Both were provided an ad libitum supply of live cotton aphids, *Aphis gossypii* Glover, (Homoptera, Aphididae) on cotton leaves, and water-soaked cotton ball was supplied as a water supplement. The boxes lined with filter paper disks. The boxes containing adults were checked daily for oviposition. If eggs were found, the adults were transferred to new plastic insect boxes (23 cm long × 15 cm wide × 9 cm high) provided with *A. gossypii* and water. The boxes containing eggs were checked daily for hatch. After the eggs had hatched and the larvae dispersed from the egg clusters, members of the F_1_ generation were placed individually into separate glass scintillation vials (20 mm diameter, 70 mm high), and reared to the desired developmental life stages. Glass-vial bioassay toxicity tests were performed using 3d old larvae, 3d pupae and 5d adults of *H. axyridis* and *C. sinica*.

All *S. manilae* and the *T. remus* populations were provided by the College of Natural Resources and Environment, South China Agricultural University, Guangzhou, China. *S. manilae* adults were stored in plastic insect boxes (23 cm long × 15 cm wide × 9 cm high) with 50–60 adults per box, and *T. remus* were stored in 30 mL glass test tubes with 200–300 adults per tube. Both were provided with honey solution and maintained at 27 ± 1 °C with a photoperiod of 12:12 h (L: D). 5 pairs of *S. manilae* adults were introduced into plastic insect boxes with 2nd instar 100–150 *S. exigua* larvae. 100–200 *T. remus* adults were introduced into a glass test tube containing *S. exigua* 2000–3000 eggs. Parasitized *S. exigua* larvae or eggs were maintained under laboratory conditions of 27 ± 1 °C, 60–75% relative humidity and photoperiod of 12:12 h (L: D). 3d old larvae and 3d old pupae of *S. manilae* and *T. remus* were used in the insect-dip method, and 5d old *S. manilae* and *T. remus* adults were used in the glass-vial bioassay.

### Insecticides

Nine insecticides were selected because of their use for control of *S. exigua*: emamectin benzoate (90%; Nanjing Redsun, China); spinosad (90%; Dow AgroSciences, China); indoxacarb (94%; DuPont, China); chlorpyrifos (97%; Dow AgroSciences, China); alpha-cypermethrin (99%; Shandong Dacheng Pesticide, China); tebufenozide (95%; Dow AgroSciences, China); chlorfenapyr (94.5%; BASF Aktiengesellschaft, China); chlorantraniliprole (95.3%; DuPont, China); methomyl (98%; Jiangsu Changlong Chemicals, China).

### Toxicity bioassays

The glass-vial bioassay^34^ was used to determine the toxicity of the insecticides to adults of *S. manilae*. Each insecticide was applied by pipetting 0.5 mL insecticide dissolved in acetone (analytical reagent, purity ≥99.7%) into each 22 mL glass scintillation vial (20 mm diameter, 70 mm height). Serial dilutions were used to obtain desired concentrations. Each vial was rolled for several minutes until an even layer of insecticide dried on the inner surface. Control treatment vials only received 0.5 mL of acetone. Vials were used the same day they were coated with the insecticides. All 9 insecticides were used the same method. Ten *S. manilae* adults were transferred into one vial then the vial was sealed with a layer of gauze. There were three (n = 3) replications were used for each rate of insecticide. After 1 h of exposure, the adults were transferred into insecticide-free vials and supplied with 10% honey solution. After 24 h, the number of dead adult *S. manilae* were counted, and the dose response (LC_50_) was calculated for each insecticide. This procedure was used for *H. axyridis* adults, *C. sinica* adults, *H. axyridis* larval, *C. sinica* larval and *T. remus* adults, however only 2 *H. axyridis* or *C. sinica* adults, 1 *H. axyridis* or *C. sinica* larva, and 10 *T. remus* adults were used per vial.

Insectcide toxicity to *S. manilae* and *T. remus* larvae was tested by the insect-dip method. A 100 ml stock solution [diluted with 5% (v/v) acetone in a water solution mixed uniformly with 5% (v/v) Tween-80] was prepared for each insecticide. Serial dilutions were used to obtain desired concentrations. 3d old *S. manilae* and *T. remus* larvae with their host were dipped for 3 s in an insecticide solution, placed on filter paper, and then individuals were transferred to separate untreated glass scintillation vials, 1 individual was used per vial. For control test, individuals were dipped in distilled water containing 5% acetone. The parasitized *S. exigua* larvae and parasitized *S. exigua* eggs were used to test the insectcide toxicity (direct) to *S. manilae* and *T. remus* larvae. The pupation rate and emergence rate of *S. manilae* and the emergence rate of *T. remus* were computed after two week to enable them to reach adulthood. The insecticide effects on *S. manilae, T. remus, H. axyridis* and *C. sinica* pupae was determined using the insect-dip method. A 100 ml stock solution [diluted with 5% (v/v) acetone in water] was prepared for each insecticide with the recommended field rate (emamectin benzoate: 3.33 mg a.i. liter^−1^; spinosad: 22.22 mg a.i. liter^−1^; indoxacarb: 33.33 mg a.i. liter^−1^; chlorpyrifos: 888.89 mg a.i. liter^−1^; alpha-cypermethrin: 33.33 mg a.i. liter^−1^; tebufenozide: 277.78 mg a.i. liter^−1^; Chlorfenapyr: 111.11 mg a.i. liter^−1^; chlorantraniliprole: 33.33 mg a.i. liter^−1^; methomy: 388.89 mg a.i. liter^−1^), the recommended field rate was obtained from the e-Pesticide Manual of ICA, MOA, China (http://www.ny100.cn/). Pupae of *H. axyridis,* pupae of *T. remus* with *S. exigua* eggshells, pupae with cocoon of *S. manilae* and *C. sinica* were dipped for 3 s in an insecticide solution, placed on filter paper, and then individuals were transferred to separate untreated glass scintillation vials, 10 individuals were used per vial. For control test, individuals were dipped in distilled water containing 5% acetone. The emergence rate of *S. manilae* and *T. remus* were computed after one week to enable them to reach adulthood.

All bioassays had 3 replications of 6–9 different insecticide concentrations, and each replication of each concentration included 20 individuals.

### Residue determination

A 100 ml stock solution [diluted with 5% (v/v) acetone in a water solution mixed uniformly with 5% (v/v) Tween-80] was prepared for each insecticide with the recommended field rate. Three pots of cabbage at adult plant stage with leaves blade (ca. 10.0 × 7.0 cm) were grouped and sprayed with insecticide until the plants were completely saturated with the solution. Treated plants were placed outside the greenhouse. All nine insecticides were tested for residue toxicity. Cabbage leaves (ca. 10.0 × 7.0 cm) were collected 0, 1, 2, 4, 7, 14, 21, 28, 35 and 42d post insecticide treatment, rinsing 4 times with Acetone, 10 ml each time, after concentrated in a blowing instrument at 40 °C, added methanol to 1 ml. The concentration of insecticide residue was determined by high performance liquid chromatography (HPLC), using a 5 um Hypersil C18 250*4.6 mm reversed phase column (Diamonsil, America). The mobile phases were methanol:acetonitrile:water (45: 50:5, v/v/v) for emamectin-benzoate and spinosad, methanol:water (80:20, v/v) for indoxacarb, tebufenozide and chlorantraniliprole, methanol:water (90:10, v/v) for chlorpyrifos, alpha-cypermethrin and chlorfenapyr, methanol:water (80:20, v/v) for indoxacarb and methanol:water (50:50, v/v) for methomyl, respectively. The detections were performed at 245 nm for emamectin-benzoate, at 252 nm for spinosad, at 234 nm for indoxacarb, at 289 nm for chlorpyrifos, at 230 nm for alpha-cypermethrin, at 240 nm for tebufenozide, at 261 nm for chlorfenapyr, at 264 nm for chlorantraniliprole and at 234 nm for methomyl. The flow rate was 0.8 ml/min. Ten μl of test solution was injected into the HPLC system.

The residue dynamics calculated by the residues/retention of water on cabbage leaves. The residues measured by HPLC = D × E × F/G × 1 ml. D is the tested insecticides volume; E is the peak area of the tested insecticides; F is the standard sample concentration; G is the peak area of the standard sample.

### Statistical analysis

LC_50_ and LC_95_ values and slopes were determined by probit analysis using the SPSS program. Survival, mortality, pupation and emergence rates were subjected to arcsine transformation and subsequently analyzed by one-way ANOVA. Means were separated by using Tukey’s Student range test (HSD) at P = 0.05 (SPSS13.0 (SPSS Inc, Chicago, USA)).

## Additional Information

**How to cite this article**: Liu, Y. *et al*. Toxicity of nine insecticides on four natural enemies of *Spodoptera exigua. Sci. Rep.* **6**, 39060; doi: 10.1038/srep39060 (2016).

**Publisher's note:** Springer Nature remains neutral with regard to jurisdictional claims in published maps and institutional affiliations.

## Figures and Tables

**Table 1:** Toxicity of insecticides on *H. axyridis, C. sinica* and *T. remus* larvae and *H. axyridis, C. sinica, S. manilae* and *T. remus* adult.

Natural enemy		Insecticides	Slope ± SE	LC_50_ (mg a.i. liter^−1^)	95% confidence limits	RQ^a^	Category^b^
*H. axyridis*	Larvae	emamectin benzoate	1.68 ± 0.20	13.31	10.75~16.86	0.17	1
		spinosad	1.63 ± 0.20	6.11	4.90~7.92	2.45	1
		indoxacarb	2.06 ± 0.23	0.033	0.027~0.040	681.82	2
		chlorpyrifos	2.20 ± 0.25	2.06	1.72~2.56	291.26	2
		alpha-cypermethrin	1.89 ± 0.21	0.61	0.50~0.75	36.89	1
		tebufenozide	1.79 ± 0.21	0.13	0.11~0.16	1442.31	2
		chlorfenapyr	2.18 ± 0.23	11.07	9.30~13.38	6.78	1
		chlorantraniliprole	2.54 ± 0.28	4.36	3.69~5.30	5.16	1
		methomyl	2.06 ± 0.23	0.015	0.012~0.018	17500	3
	Adult	emamectin benzoate	1.87 ± 0.21	78.95	64.74~99.04	0.028	1
		spinosad	2.03 ± 0.22	32.26	26.77~39.86	0.46	1
		indoxacarb	1.43 ± 0.19	43.1	33.18~55.16	0.52	1
		chlorpyrifos	2.38 ± 0.24	0.04	0.034~0.048	15000	3
		alpha-cypermethrin	1.71 ± 0.21	1.91	1.53~2.51	11.78	1
		tebufenozide	1.37 ± 0.19	0.48	0.37~0.68	390.63	2
		chlorfenapyr	1.40 ± 0.20	0.98	0.75~1.39	76.53	2
		chlorantraniliprole	1.93 ± 0.21	57.88	47.78~70.69	0.39	1
		methomyl	2.01 ± 0.21	51.12	42.39~61.75	5.13	1
*C. sinica*	Larvae	emamectin benzoate		>3.33		<1	1
		spinosad		>22.22		<1	1
		indoxacarb		>33.33		<1	1
		chlorpyrifos		>888.89		<1	1
		alpha-cypermethrin		>33.33		<1	1
		tebufenozide		>277.78		<1	1
		chlorfenapyr		>111.11		<1	1
		chlorantraniliprole		>33.33		<1	1
		methomyl		>388.89		<1	1
	Adult	emamectin benzoate	1.94 ± 0.21	0.24	0.20~0.29	9.38	1
		spinosad	1.55 ± 0.19	22.77	17.91~28.71	0.66	1
		indoxacarb	2.12 ± 0.22	0.029	0.025~0.035	775.86	2
		chlorpyrifos	1.71 ± 0.20	0.63	0.51~0.78	952.38	2
		alpha-cypermethrin	1.72 ± 0.20	1.81 × 10^−3^	1.44 × 10^−3^ ~2.24 × × 10^−3^	12430.9	3
		tebufenozide	1.87 ± 0.21	34.16	27.92~43.31	5.49	1
		chlorfenapyr	1.90 ± 0.21	6.00 × 10^−5^	5.00 × 10^−5^~7.00 × 10^−5^	1.25 × 10^6^	3
		chlorantraniliprole	1.98 ± 0.21	29.09	24.11~35.35	0.77	1
		methomyl	1.94 ± 0.21	2.40 × 10^−3^	1.97 × 10^−3^ ~2.90 × 10^−3^	1.33 × 10^5^	3
*S. manilae*	Adult	emamectin benzoate	2.45 ± 0.24	3.00 × 10^−4^	2.60 × 10^−4^~3.60 × 10^−4^	7500	3
		spinosad	2.51 ± 0.24	5.76 × 10^−3^	4.92 × 10^−3^~6.78 × 10^−3^	2604.17	3
		indoxacarb	0.98 ± 0.14	0.084	0.052~0.17	267.86	2
		chlorpyrifos	2.33 ± 0.24	0.071	0.060~0.085	8450.7	3
		alpha-cypermethrin	2.55 ± 0.26	3.20 × 10^−5^	2.73 × 10^−5^~3.79 × 10^−5^	7.03 × 10^5^	3
		tebufenozide	1.18 ± 0.13	9.35 × 10^−5^	6.78 × 10^−5^~1.28 × 10^−4^	2.01 × 10^6^	3
		chlorfenapyr	2.15 ± 0.22	1.05 × 10^−3^	8.80 × 10^−4^~1.26 × 10^−3^	71428.6	3
		chlorantraniliprole	2.09 ± 0.22	2.75	2.30~3.35	8.18	1
		methomyl	2.61 ± 0.28	4.81 × 10^−6^	4.10 × 10^−6^~5.76 × 10^−6^	5.46 × 10^7^	3
*T. remus*	Larvae	emamectin benzoate		>3.33		<1	1
		spinosad		>22.22		<1	1
		indoxacarb		>33.33		<1	1
		chlorpyrifos		>888.89		<1	1
		alpha-cypermethrin		>33.33		<1	1
		tebufenozide		>277.78		<1	1
		chlorfenapyr		>111.11		<1	1
		chlorantraniliprole		>33.33		<1	1
		methomyl		>388.89		<1	1
	Adult	emamectin benzoate		<1 × 10^−9^		>2.25 × 10^9^	3
		spinosad		<1 × 10^−7^		>1.50 × 10^8^	3
		indoxacarb		<1 × 10^−9^		>2.25 × 10^10^	3
		chlorpyrifos		<1 × 10^−9^		>6.00 × 10^11^	3
		alpha-cypermethrin		<1 × 10^−12^		>2.25 × 10^13^	3
		tebufenozide		<1 × 10^−10^		>1.88 × 10^12^	3
		chlorfenapyr		<1 × 10^−6^		>7.50 × 10^7^	3
		chlorantraniliprole		<1 × 10^−5^		>2.25 × 10^6^	3
		methomyl		<1 × 10^−7^		>2.63 × 10^9^	3

RQ^a^, risk quotient = recommended field rate (g a.i. ha^−1^)/LC_50_ of *H. axyridis, C. sinica* and *T. remus* larvae and *H. axyridis, C. sinica, S. manilae* and *T. remus* adult (mg mg a.i. liter^−1^).

Category^b^, 1: safe; 2: slightly to moderately toxic; 3: dangerously toxic.

**Table 2:** Toxicity of insecticides on pupae of *H. axyridis, C. sinica, S. manilae* and *T. remus.*

Insecticides	The recommended field rate (mg a.i. liter^−1^)	Emergence rate (%)			
		*H. axyridis* (±SD) (%)	*C. sinica* (±SD) (%)	*S. manila* (±SD) (%)	*T. remus* (±SD) (%)
emamectin benzoate	3.33	94.44 ± 2.22 a	93.33 ± 1.93 a	96.67 ± 1.93 a	95.56 ± 2.94 a
spinosad	22.22	92.22 ± 1.11 a	94.44 ± 1.11 a	97.78 ± 1.11 a	93.33 ± 1.93 a
indoxacarb	33.33	95.56 ± 1.11 a	94.44 ± 2.93 a	96.67 ± 1.93 a	94.44 ± 1.11 a
chlorpyrifos	888.89	LC_50_: 527.68 mg·L^−1^	93.33 ± 1.93 a	95.56 ± 1.11 a	96.67 ± 3.33 a
alpha-cypermethrin	33.33	97.78 ± 1.11 a	97.78 ± 1.11 a	97.78 ± 1.11 a	94.45 ± 4.01 a
tebufenozide	277.78	LC_50_: 350.08 mg·L^−1^	95.56 ± 1.11 a	95.56 ± 1.11 a	94.45 ± 2.22 a
chlorfenapyr	111.11	94.44 ± 2.94 a	93.33 ± 3.33 a	95.55 ± 2.22 a	94.44 ± 1.11 a
chlorantraniliprole	33.33	94.44 ± 1.11 a	92.22 ± 1.11 a	96.67 ± 1.93 a	96.67 ± 1.93 a
methomyl	388.89	LC_50_: 314.75 mg·L^−1^	93.33 ± 1.93 a	95.56 ± 1.11 a	95.56 ± 4.44 a
control		95.56 ± 1.11 a	94.44 ± 1.11 a	96.67 ± 1.93 a	95.56 ± 1.11 a

The data in the table are mean ± SE, and those in the same column followed by same letters are not significantly different (*P* < 0.05).

**Table 3:** Toxicity of insecticides on *S. manilae* larvae.

Insecticides	Concentration (mg a.i. liter^−1^)	Survival Rate (±SD) (%) of *S. exigua* (48 h)	Pupation rate (±SD) (%)	Emergence rate (±SD) (%)
emamectin benzoate	0.022	41.33 ± 2.96	94.84 ± 2.60 a	94.41 ± 2.83 a
spinosad	0.97	51.33 ± 2.96	93.86 ± 3.41 a	95.40 ± 2.31 a
indoxacarb	0.15	37.67 ± 2.33	94.44 ± 5.56 a	97.22 ± 2.78 a
chlorpyrifos	0.063	44.33 ± 4.67	100 ± 0.0 a	95.35 ± 2.36 a
alpha-cypermethrin	1.21	37.67 ± 3.93	94.87 ± 5.13 a	97.22 ± 2.78 a
tebufenozide	112.23	52.33 ± 2.91	94.00 ± 3.40 a	97.78 ± 2.22 a
chlorfenapyr	7.56	44.33 ± 2.96	95.21 ± 2.41 a	97.62 ± 2.38 a
chlorantraniliprole	0.103	36.67 ± 2.03	94.19 ± 2.91 a	96.97 ± 3.03 a
methomyl	48.32	38.00 ± 1.00	94.19 ± 2.91 a	96.97 ± 3.03 a
control		98.00 ± 1.00	94.33 ± 1.33 a	98.81 ± 1.19 a

The data in the table are mean ± SE, and means followed by the same letter are not significantly different; LSD, *P* < 0.05.

## References

[b1] Smagghe G. . Toxicity and kinetics of methoxyfenozide in greenhouse-selected *Spodoptera exigua* (Lepidoptera: Noctuidae). Pest Manage. Sci. 59, 1203–1209 (2003). 10.1002/ps.756 14620046

[b2] Metcalf R. L. & Metcalf R. A. Destructive and useful insects : 5th ed. McGraw-Hill, New York (1992).

[b3] Zheng X. L., Cong X. P., Wang X. P. & Lei C. L. A review of geographic distribution, overwintering and migration in Spodoptera exigua (Hübner) (Lepidoptera: Noctuidae). J. Entomol. Res. Soc. 13, 39–48 (2011).

[b4] Wang Y. H. . Susceptibility of adult *Trichogramma nubilale* (Hymenoptera: Trichogrammatidae) to selected insecticides with different modes of action. Crop Prot. 34, 76–82 (2012).

[b5] Fogel M. N., Schneider M. I., Desneux N., González B. & Ronco A. E. Impact of the neonicotinoid acetamiprid on immature stages of the predator *Eriopis connexa* (Coleoptera: Coccinellidae). Ecotoxicology 22, 1063–1071 (2013). 23793295 10.1007/s10646-013-1094-5

[b6] Bacci L. . Toxicity of insecticides to the sweetpotato whitefly (Hemiptera: Aleyrodidae) and its natural enemies. Pest Manage. Sci. 63, 699–706 (2007). 10.1002/ps.1393 17523144

[b7] Preetha G., Stanley J., Suresh S. & Samiyappan R. Risk assessment of insecticides used in rice on miridbug, *Cyrtorhinus lividipennis* Reuter, the important predator of brown planthopper, *Nilaparvata lugens* (Stal). Chemosphere 80, 498–503 (2010). 20537680 10.1016/j.chemosphere.2010.04.070

[b8] Wright D. J. & Verkert R. H. J. Integration of chemical and biological control systems for arthropods; evaluation in a multitrophic context. Pest Manage. Sci. 44, 207–218 (1995).

[b9] Wang H. Y. . Assessment of the impact of insecticides on *Anagrus nilaparvatae* (Pang et Wang) (Hymenoprera: Mymanidae), an egg parasitoid of the rice planthopper, *Nilaparvata lugens* (Hemiptera: Delphacidae). Crop Prot. 27, 514–522 (2008).

[b10] Greathead D. J. Natural enemies in combination with pesticides for integrated pest management. In Reuveni R. ed Novel Approaches to Integrated Pest Management Lewis Publishers, Boca Raton, FL, USA 183–197 (1995).

[b11] Brown P. M. J. . The global spread of *Harmonia axyridis* (Coleoptera: Coccinellidae): distribution, dispersal and routes of invasion. Biocontrol 56, 623–641 (2011).

[b12] Xu Y. Y., Mou J. Y. & Hu C. Research and application of *Chrysoperla sinica* (Tjeder). Entomol. Knowl. 36, 313–315 (1999).

[b13] Sun J. S. & Huang S. S. Evaluation of potential control ability of *Snellenius manilae* (Ashmead) against *Spodoptera exigua* (Hübner). *Acta Ecol. Sin*. 30, 1494–1499 (2010).

[b14] Si S. Y. . Progress in research on prevention and control of beet armyworm, *Spodoptera exigua* in China. Chin. J. Appl. Entomol. 49, 1432–1438 (2012).

[b15] Cave R. D. Biology, ecology and use in pest management of *Telenomus remus*. Biocontr. News Inform. 21, 21–26 (2000).

[b16] Yang Y., Han Y., Fang Z. H. & Xu Z. F. Effect of host egg age and contact time on the parasitic capacity of *Telenomus remus* (Hymenoptera: Scelionidae). Chin. J. Appl. Entomol. 49, 1490–1495 (2012).

[b17] Lu Y. H., Wu K. M., Jiang Y. Y., Guo Y. Y. & Desneux N. Widespread adoption of Bt cotton and insecticide decrease promotes biocontrol services. Nature 487, 362–367 (2012). 22722864 10.1038/nature11153

[b18] Wyckhuys K. A. G. . Current status and potential of conservation biological control for agriculture in the developing world. Biol. Control 65, 152–167 (2013).

[b19] Desneux N., Decourtye A. & Delpuech J. M. The sublethal effects of pesticides on beneficial arthropods. Annu. Rev. Entomol. 52, 81–106 (2007). 16842032 10.1146/annurev.ento.52.110405.091440

[b20] Lahm G. P., Cordova D. & Barry J. D. New and selective ryanodine receptor activators for insect control. Bioorg. Med. Chem. 17, 4127–4133 (2009). 19186058 10.1016/j.bmc.2009.01.018

[b21] Cordova D. . Anthranilic diamides: a new class of insecticides with a novel mode of action, ryanodine receptor activation. Pestic. Biochem. Phys. 84, 196–214 (2006).

[b22] Brugger K. E. . Selectivity of chlorantraniliprole to parasitoid wasps. Pest Manag. Sci. 66, 1075–1081 (2010). 20540073 10.1002/ps.1977

[b23] Tomlin C. The Pesticide Manual, 13th ed. British Crop Protection Council, Cambridge, UK, 1344 (2003).

[b24] Giraddi R. S. & Gundannavar K. P. Safety of emamectin benzoate, an avermectin derivative to the egg parasitoids, *Trichogramma* spp. Karnataka J. Agric. Sci. 19, 417–418 (2006).

[b25] Xu H. H. Plant chemical protection, pp. 72–115. China Agriculture Press, Beijing (2007).

[b26] Wang Y. H. . Susceptibility of adult *Trichogramma nubilale* (Hymenoptera: Trichogrammatidae) to selected insecticides with different modes of action. Crop Prot. 34, 76–82 (2012).

[b27] Schoonover J. R. & Larson L. L. Laboratory activity of spinosad on non-target beneficial arthropods, 1994. Arthrop. Manag. Tests 20, 357 (1995).

[b28] Gerling D. & Sinai P. Buprofezin effects on two parasitoid species of whitefly (Homoptera:Aleyrodidae). J. Econ. Entomol. 87, 842–846 (1994).

[b29] Jones W. A., Ciomperlik M. A. & Wolfenbarger D. A. Lethal and sublethal effects of insecticides on two parasitoids attacking *Bemisia argentifolii* (Homoptera, Aleyrodidae). Biol. Control 11, 70–76 (1998).

[b30] Van Lenteren J. C. Success in biological control of arthropods by augmentation of natural enemies. In Gurr G., Wratten S. eds Measures of Success in Biological Control. Kluwer Acadamic Publishers, Dordrecht, Netherlands, 77–103 (2000).

[b31] Murthy K. S., Rao N. S., Rabinfra R. J. & Jalali S. K. Age related parasitisation potential of the eggs parasitoid *Telenomus remus* (Scelionidae: Hymenoptera) on certain lepidopterous hosts. J. Entomol. Res. 28, 33–36 (2004).

[b32] Tang Y. L., Chen K. W. & Xu Z. F. Study on ontogenesis of *Telenomus remus* Nixon (Hymenoptera: Scelionidae). J. Changjiang Vege. 18, 1–3 (2010).

[b33] Che W. N. Shi T. Wu Y. D. & Yang Y. H. Insecticide resistance status of field populations of *Spodoptera exigua* (Lepidoptera: Noctuidae) from China. J. Econ. Entomol. 106, 1855–1862 (2013). 24020303 10.1603/ec13128

[b34] Snodgrass G. L. Glass-vial bioassay to estimate insecticide resistance in adult tarnished plant bugs (Heteroptera: Miridae). J. Econ. Entomol. 89, 1053–1059 (1996).

